# Risk Model Assessment in Early-Onset and Adult-Onset Schizophrenia Using Neurological Soft Signs

**DOI:** 10.3390/jcm8091443

**Published:** 2019-09-11

**Authors:** Bao-Yu Chen, I-Ning Tsai, Jin-Jia Lin, Ming-Kun Lu, Hung-Pin Tan, Fong-Lin Jang, Shu-Ting Gan, Sheng-Hsiang Lin

**Affiliations:** 1 Institute of Clinical Medicine, College of Medicine, National Cheng Kung University, 35, Xiaodong Rd., North Dist., Tainan City 70457, Taiwan; totalmicchen@hotmail.com (B.-Y.C.); 0312elise@gmail.com (I.-N.T.);; 2 Department of Psychiatry, Chi Mei Medical Center, 901, Zhonghua Rd. Yongkang Dist., Tainan City 71004, Taiwan; jinjialin5483@gmail.com (J.-J.L.); jfl5124@gmail.com (F.-L.J.); 3 Department of Health, Jianan Mental Hospital, 539, Yuzhong Rd., Rende Dist., Tainan City 71742, Taiwan; msdrkun@yahoo.com.tw (M.-K.L.); bed100@msn.com (H.-P.T.); 4 Department of Applied Life Science and Health, Chia Nan University of Pharmacy and Science, 60, Sec. 1, Erren Rd., Rende Dist., Tainan City 71710, Taiwan; 5 Department of Environmental and Occupational Health, College of Medicine, National Cheng Kung University, 35, Xiaodong Rd., North Dist., Tainan City 70457, Taiwan; 6 Department of Public Health, College of Medicine, National Cheng-Kung University, 35, Xiaodong Rd., North Dist., Tainan City 70457, Taiwan; 7 Biostatistics Consulting Center, National Cheng Kung University Hospital, College of Medicine, National Cheng Kung University, 35, Xiaodong Rd., North Dist., Tainan City 70457, Taiwan

**Keywords:** schizophrenia, endophenotype, neurodevelopmental markers, neurological soft signs, familial aggregation, recurrence risk ratio

## Abstract

Age at onset is one of the most important clinical features of schizophrenia that could indicate greater genetic loadings. Neurological soft signs (NSS) are considered as a potential endophenotype for schizophrenia. However, the association between NSS and different age-onset schizophrenia still remains unclear. We aimed to compare risk model in patients with early-onset schizophrenia (EOS) and adult-onset schizophrenia (AOS) with NSS. This study included 262 schizophrenia patients, 177 unaffected first-degree relatives and 243 healthy controls. We estimated the discriminant abilities of NSS models for early-onset schizophrenia (onset age < 20) and adult-onset schizophrenia (onset age ≥ 20) using three data mining methods: artificial neural networks (ANN), decision trees (DT) and logistic regression (LR). We then assessed the magnitude of NSS performance in EOS and AOS families. For the four NSS subscales, the NSS performance were greater in EOS and AOS families compared with healthy individuals. More interestingly, there were significant differences found between patients’ families and control group in the four subscales of NSS. These findings support the potential for neurodevelopmental markers to be used as schizophrenia vulnerability indicators. The NSS models had higher discriminant abilities for EOS than for AOS. NSS were more accurate in distinguishing EOS patients from healthy controls compared to AOS patients. Our results support the neurodevelopmental hypothesis that EOS has poorer performance of NSS than AOS. Hence, poorer NSS performance may be imply trait-related NSS feature in EOS.

## 1. Introduction

Neurological soft signs (NSS) represent neurodevelopmental abnormalities which occur at higher frequencies among schizophrenia patients than in healthy individuals [1,2,3]. A brain imaging study confirmed that NSS are strongly associated with regional neuroanatomical characteristics and concluded that physical signs are markers for malformed brain development [4]. Previous studies have suggested that NSS are potential endophenotype encompassing genetic and non-genetic processes in the neurodevelopment of schizophrenia [5,6]. Further, robust evidence has shown that relatives of schizophrenia patients are more affected by NSS than healthy individuals (but less affected than schizophrenia patients) [7] and are sensitive to the development of psychosis [8]. Some studies also supported the trait aspect of NSS and suggested the NSS as a potential endophenotype or marker which contain genetic liability for psychosis [9,10]. Therefore, investigating NSS in schizophrenia patients with different onset ages and their first-degree relatives could contribute much to understanding underlying genetic load in different age-onset schizophrenia.

Schizophrenia usually begins in late adolescence or young adulthood and age at onset is an essential clue in determining the disease’s pathogenesis. Previous studies have reported that early-onset schizophrenia (EOS) is associated with a poorer prognosis and tendency to resist treatment [11,12,13,14]. A systematic review showed that EOS patients have worse outcomes than AOS patients, including greater symptom severity, social withdrawal, suicide and negative cognitive impacts [15]. Therefore, age of onset may be a critical indicator of poor outcomes during neurodevelopment. A previous study supported the assertion that NSS were significantly more frequent in child- and adolescent-onset compared to adult- onset schizophrenia [16]. However, this study lacked adequate healthy controls and had a limited sample size. Another longitudinal study of early-onset first-episode psychosis had more patients with NSS than healthy controls, suggesting that these signs may be considered trait markers [17]. Thus, schizophrenia patients with different onset ages may have various degrees of NSS, and may also be influenced by genetic and environmental factors.

A meta-analysis confirmed that there are differences in NSS between schizophrenia patients, their non-psychotic relatives and healthy individuals [18]. Relatives of schizophrenia patients showed significantly more NSS than healthy controls, but fewer than schizophrenia patients [19]. Interestingly, a review study reported that the relationships between temperament, character features and NSS aggregate in the relatives of schizophrenia patients, which suggests there is genetic vulnerability to schizophrenia [20]. A recent study revealed that the Sensory Integration (SI) and the Motor Coordination (MC) of NSS subscales showed high estimated heritability between schizophrenia patients and their non-psychotic first-degree relatives [21]. Based on these findings, we hypothesise that there may be different magnitudes of NSS performance among EOS patients, AOS patients and their first-degree relatives.

The present study uses novel hybrid classification approaches such as data mining algorithms (e.g., artificial neural networks and decision trees) and logistic regressions to improve the validity of NSS models in EOS and AOS patients. To strengthen sampling and compare model performance, 10-fold cross-validation methods are used to measure the unbiased estimates of each model. Second, this study assesses the magnitude of NSS performance in EOS and AOS patients’ non-psychotic first-degree relatives. Therefore, we hypothesise that EOS, AOS and their first-degree relatives result in poorer NSS performance than healthy controls. Likewise, we suggest that NSS are significantly more frequent in patients whose schizophrenia began in childhood or adolescence compared to patients whose schizophrenia began in adulthood.

## 2. Materials and Methods

### 2.1. Participants

Schizophrenia patients were recruited from the inpatient wards and outpatient clinics of three medical institutions in southern Taiwan: the Chi Mei Medical Center, the Jianan Mental Hospital and the Lok An Hospital. Data was collected from April 2011 to March 2016. Patients were included in the study if they met the Diagnostic and Statistical Manual of Mental Disorders, Fourth Edition (DSM-IV-TR) criteria for schizophrenia. All recruited patients are considered by the psychiatrists as stabilized. The nonpsychotic first-degree relatives of the patients were also recruited for the study. For comparison with healthy controls, members of hospital staff and community without a past history of any psychiatric disorder were recruited as well. All participants aged 20 years or older were included in the present study. Written informed consent was obtained from all subjects after complete description of the study. All participants were Taiwanese Han Chinese in ethnic origin. This study included 262 schizophrenia patients, 177 first-degree relatives of schizophrenia patients with no psychotic symptoms and 243 healthy individuals. All participants were interviewed were carried out by a well-trained research assistant with standardized psychiatric interviewing training and more than decade psychiatric ward experience using the Chinese version of the Diagnostic Interview for Genetic Studies (DIGS), which is a clinical interview particularly constructed for the assessment of major mood and psychotic disorders and their spectrum conditions [22,23]. This is a structured interview used in studies on psychiatric disorders to confirm the suitability of potential study subjects. The establishment of the Chinese version of the DIGS and its reliability has been described elsewhere [22]. The exclusion criteria were: (1) a history of illegal substance or alcohol abuse, identifiable neurological disorders, brain surgery, mental retardation, or somatic disorders with neurological components; or (2) having a parent who was not Han Chinese. The study’s design and recruitment procedures received ethics approval by the institutional review boards (IRBs) of participating hospitals (IRB numbers: 10301-002, 11-011, and B-BR-103-036-T).

### 2.2. Age at Onset of Schizophrenia

Many previous studies showed that the symptoms need to be diagnosed before the patient is 20 years old for EOS [24,25,26]. However, a different cut-off point may influence the study results. The study used different cut-off points for the age of onset to identify the best cut-off point. A meta-analysis study also suggested that the gender difference in age at onset of schizophrenia is smaller than previously thought [27]. Therefore, schizophrenia patients were divided into two subgroups according to onset age: EOS patients whose onset of symptoms occurred before the age of 20 and AOS patients whose onset occurred after the age of 20. Within the limits of recalling lifetime events, delays in initial mental health care may lead to information bias for onset age. Thus, the age of onset in current study was defined via consulting patients’ family members and tracking the medical records.

### 2.3. Measurements 

#### 2.3.1. Assessment of Neurological Soft Signs (NSS) and Craniofacial Features

NSS are minor neurological abnormalities that we evaluated using the Neurological Evaluation Scale (NES) by Buchanan and Heinrichs [1]. A patient’s minor neurological abnormalities are divided into four subscales with a total of 26 items: (1) Sensory Integration (e.g., finger agnosia and extinction), (2) Motor Coordination (e.g., finger opposition and rapid finger tapping), (3) Sequencing of Complex Motor Acts (e.g., fist-ring, fist-edge-palm, Ozeretski test and rhythm tapping test) and (4) Others (e.g., adventitious overflow, Romberg test, Romberg: tremor, memory 5 min/10 min, mirror movements, synkinesis, convergence and gaze impersistence). Each item was rated on a 3-point scale: *absent* (0), *mild* (1) and *impairment* (2) by trained researchers. The NSS scale measuring procedure was established by a neurologist at the Chi Mei Medical Centre. The two research members (a PhD student and a research assistant) were both well-trained by a neurologist to perform the NSS assessment. The intraclass correlation measures the reliability of items ratings are ranged from 0.77 to 1.0.

#### 2.3.2. Discriminant Models

Two different hybrid data mining procedures and one traditional statistical method were applied: artificial neural networks (ANN), decision trees (DT) and logistic regression (LR). ANN is a model developed in the field of computational linguistics that is based on the structure of biological neural networks. It has become firmly established as a powerful method with potential for analysing any subject, but is especially useful in the medical field [28]. In the current study, the ANN model contained three layers (input, hidden and output layers) for estimating functions. In each model, the input layer contained neurons including sex, age, body mass index (BMI) and the different NSS subscales (SI, MC, Sequencing of Complex Motor Acts and Other). In the hidden layers, the system was optimized to maximize accuracy by using training and validation data in a trial-and-error process. The output layer in each model had two neurons: cases and controls. DTs are tree-like models that use explainable logic statements (if-then statements) to show results or effects of decisions and actions [29]. We used DTs together with Classification and Regression Trees (CART) to distinguish NSS items. LR is a commonly used statistical model where the probability of the dichotomous outcome (schizophrenia patients = 1 and healthy controls = 0) is associated with the independent variables used. We used a stepwise method for variable selection in the logistic regression.

#### 2.3.3. Statistical Analysis

First, we used LR to compare the NSS scores of the EOS, AOS and HC groups and to make adjustments for sex, age and BMI. Frequency matching was used in EOS–AOS to assure that both groups have the same distributions over strata defined by DIGS-C positive and negative score. We used mixed-effect models to compare the NSS scores of the relatives of EOS and AOS patients to the HC group (we also made adjustments for sex, age and BMI). Second, we used two data mining approaches (ANN, DT) and a commonly used statistical method (LR) to construct discriminant models for NSS. In order to present unbiased estimates of the models, we used 10-fold cross-validation methods. Finally, we used relative recurrence-risk ratios to estimate the magnitude of familial aggregation at different NSS cut-off scores. The λ coefficient was calculated as the recurrence risk ratio among relatives compared to the prevalence in the general population:

$$\lambda =\frac{\mathrm{Pr}\;(\mathrm{relatives}\;\mathrm{with}\;\mathrm{NSS}\;|\;\mathrm{having}\;a\;\mathrm{proband}\;\mathrm{with}\;\mathrm{NSS})}{\mathrm{Pr}\;(\mathrm{general}\;\mathrm{population}\;\mathrm{with}\;\mathrm{NSS})}$$

Thus, we used different NSS cut-off scores to determine the recurrence risk ratios according to different ages at onset. We performed ANN, DT and LR modelling using SAS Enterprise Miner version 14.1. The remaining statistical analyses were conducted using SAS Statistical Software, version 9.4 (SAS Institute, Cary, NC, USA).

## 3. Results

### 3.1. Descriptive Data

We recruited 205 patients with schizophrenia for this study. This group included 82 early-onset schizophrenia (EOS) patients and 123 adult-onset schizophrenia (AOS) patients. We also recruited 134 non-psychotic relatives, including 53 relatives of EOS patients and 81 relatives of AOS patients. We recruited 243 healthy individuals as controls to compare with the schizophrenia patients and their relatives. Table 1 shows participant demographic characteristics. The mean onset age of schizophrenia was 16.96 years for EOS patients and 27. 12 years for AOS patients. The mean duration of illness in EOS patients was 19.88 years, while in AOS patients it was 16.01 years. We found that mean weight and BMI were higher in both EOS and AOS schizophrenia patients compared to their relatives or healthy individuals. There were also a greater number of men among the schizophrenia patients than among non-psychotic relatives and healthy individuals.

### 3.2. Discriminant Models of Nss in Early-Onset, Adult-Onset Schizophrenia and The Nss Performance of the Schizophrenia First-Degree Relatives

**Figure 1:**
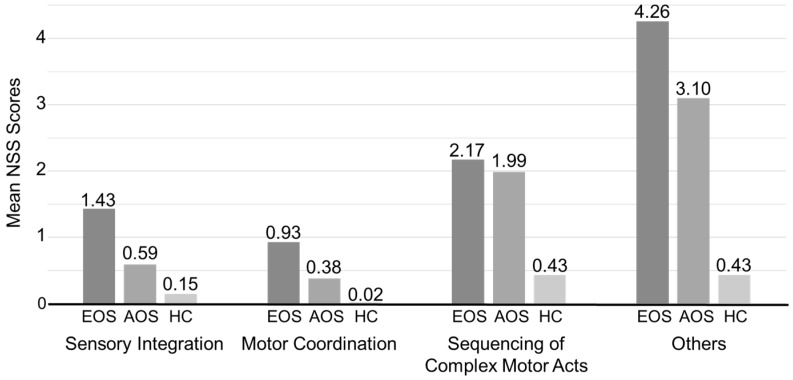
Mean neurological soft sign (NSS) scores in early-onset schizophrenia (EOS) patients, adult-onset schizophrenia (AOS) patients, and healthy controls (HC).

**Figure 2:**
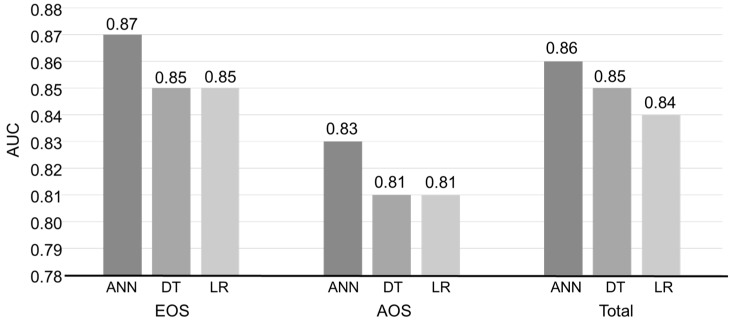
Neurological soft sign (NSS) prediction accuracy estimated via training full data and 10-fold cross-validation for early-onset (EOS) and adult-onset schizophrenia (AOS), and all patients with schizophrenia (Total). ANN, artificial neural networks; DT, decision trees; LR, logistic regression.

Throughout our careful evaluation, EOS and AOS patients’ total NSS scores were compared with HC scores (Table 2, Figure 1). For the total NSS score, the means of EOS, AOS and HC group were 9.95, 7.58 and 1.43, respectively. In the SI subscale, the mean score for EOS patients was 1.43, for AOS patients it was 0.59 and for the HC group it was 0.15. Therefore, on the SI subscale, patients with EOS or AOS had higher scores than healthy individuals (*p* < 0.0001). Similar results were observed in the MC, Sequencing of Complex Motor Acts (SCMA) and Others (O) subscales. Furthermore, the NSS scores of EOS patients were higher than those of AOS patients using the SI and MC subscales. We used both full data and 10-fold cross-validation methods to measure the unbiased estimates of our models. We tested the discriminant accuracy of the three data mining methods described above by measuring each model’s area under the curve (AUC), accuracy, sensitivity and specificity for EOS patients, AOS patients and all patients (Table 3, Figure 2). For EOS patients, ANN had the greatest discriminant ability of the three data mining methods. Table 3 shows that using the training full data, the accuracy of ANN in EOS patients, AOS patients and all patients was 84%, 78% and 82%, respectively. Using DT, ANN modelling was 83%, 80% and 82% accurate and using LR it was 82%, 78% and 79% accurate. In terms of 10-fold cross-validation, the accuracy of ANN in EOS patients, AOS patients and all patients were 84%, 78% and 82%, respectively; for DT accuracies were 82%, 80% and 81%, respectively; and LR was 81%, 76% and 76% accurate, respectively. Furthermore, the NSS-based data mining models had even higher accuracy levels when all NSS subscales were treated as input variables (Table 3). On the other hand, we examined the NSS scores of non-psychotic relatives of EOS and AOS patients in comparison to the HC group (Table 4). For the total NSS score, the mean of non-psychotic relatives of EOS and AOS were 4.51 and 4.87, respectively. All NSS subscales showed that non-psychotic relatives of EOS and AOS patients had significantly higher scores than HCs (*p* < 0.0001). However, across all NSS subscales, the differences in scores between non-psychotic relatives of EOS patients and those of AOS patients were not significant. In conclusion, across the three data mining methods, the EOS group had the highest AUC and consummate accuracy compared with the AOS group and all patients.

### 3.3. Familial Aggregation of Nss in Early-Onset Schizophrenia and Adult-Onset Schizophrenia Patient Families

**Figure 3:**
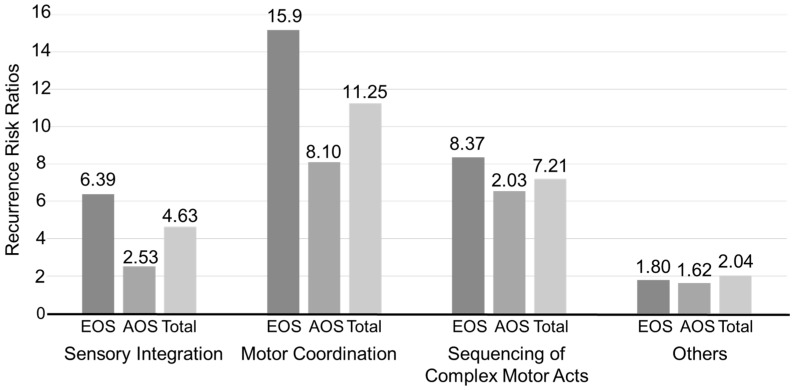
The proportion of subjects with NSS scores above cut-off thresholds and corresponding recurrence risk ratios. For Motor Coordination, the NSS cut-off point is ≥1; for Sensory Integration, Motor Coordination and Sequencing of Complex Motor Acts, the NSS cut-off point is ≥2 due to the stability of the model.

We examined the NSS scores of non-psychotic relatives of EOS and AOS patients in comparison to the HC group (Table 4). All NSS subscales showed that non-psychotic relatives of EOS and AOS patients had significantly higher scores than HCs (*p* < 0.0001). However, across all NSS subscales, the differences in scores between non-psychotic relatives of EOS patients and those of AOS patients were not significant. We then used recurrence risk ratios to estimate the familial aggregation of NSS (Figure 3). Based on their NSS scores, patients were categorized as either having NSS or not, and the data were treated as binary. Then, recurrence risk ratios were estimated for non-psychotic relatives of EOS and AOS patients based on cut-off scores for each NSS subscale. Figure 3 shows the different NSS cut-off scores for each subscale in order to provide a stable estimate. Using the highest cut-off score for SI (cut-off NSS ≥ 2), the risk ratios were 6.39 (1.73–23.58) in EOS patient families; 2.53 (0.32–19.77) in AOS patient families; and 4.63 (1.37–15.59) in all schizophrenia patients. For MC (cut-off NSS ≥ 1), the risk ratios were 15.19 (5.52–41.79) in EOS patient families; 8.10 (2.49–26.36) in AOS patient families; and 11.25 (4.35–29.08) in all schizophrenia patients. For the SCMA and Other subscales, no significant differences in risk ratios were found between EOS and AOS patient families. These findings suggest that there is familial aggregation of some NSS-associated symptoms and that EOS families have higher familial aggregation than AOS families.

## 4. Discussion

To our knowledge, this is the first study to assess familial aggregation of NSS between schizophrenia patients with different onset ages and their relatives. We used three different statistical models (e.g., ANN, DT and LR) to enhance the accuracy and validity of the NSS models for EOS and AOS patients. We found that all NSS models had higher discriminant validity for EOS patients than for AOS patients. Thus, we support that the models of NSS might be useful discriminate tools to identify different minor neurological impairments between early- and adult- onset schizophrenia. Further, EOS patients had higher scores than AOS patients on the SI and MC NSS subscales. Poorer NSS performance was also observed in first-degree relatives of schizophrenia patients than HC on all subscales. These major findings suggest that EOS and AOS patient families may be affected differently according to either genetic or shared environmental factors.

Previous studies of EOS in patients have shown that it is associated with a high premorbid prevalence of severe neurodevelopmental abnormalities and poor psychosocial outcomes [30,31]. In clinical psychiatry studies, EOS also tends to be treatment-refractory. That is, patients do not respond adequately to appropriate courses of at least two antidepressants [32]. The EOS may indicate a more severe condition of illness associated with greater genetic predisposition than AOS [33,34]. In our empirical research, no matter which algorithm method was used, the NSS models of EOS had higher accuracy and discriminant validity than AOS. These results suggest that the childhood manifestation of a typically adult-onset illness presents severe symptoms and more genetic influences on the neurodevelopmental model [35].

In the current study, we found that EOS patients had more severe SI and MC condition than AOS patients. Conventionally, sensorimotor cortex impairment has been considered a clinical neurological sign that contributes to NSS in schizophrenia patients [36]. One interesting finding from a study of first schizophrenic episodes is an association between the reduction of frontal and temporal lobe brain grey matter volumes high scores on NSS subscales, especially SI and MC [37]. These brain areas are normally tangled with auditory, attention and language processes, as well as related audio-visual integration [38]. In one recent study, the mean MC scores in healthy individuals revealed relatively fewer than schizophrenia patients [39], which is also the case in the present study.

In the majority of EOS patients, premorbid disturbances appear in the social, motor and language domains [40,41]. Therefore, we posit that SI and MC could differentiate EOS from AOS. Although impairment on these subscales occurs in both EOS and AOS patients, EOS patients had significantly higher scores. Thus, we support that the evaluation of NSS as a potential marker of age at onset in schizophrenia. A longitudinal brain imaging study supports the assertion that EOS patients have more dynamic brain changes than healthy children [42]. These results indicate earlier biological vulnerability and an extended period of irregular neurodevelopment in schizophrenia patients [43]. Our results also confirm that EOS patients have more disorders in language domains since EOS patients have more SI and MC condition than AOS patients. Consequently, the SI and MC NSS subscales may be relevant to understanding the genetic factors underlying schizophrenia.

We also observed significant differences between the non-psychotic relatives of EOS and AOS patients on the SI and MC NSS subscales. A study of schizophrenia examined the heritability and familiality of NSS by sampling twins and showed moderately high correlation with first-degree relatives [21]. The current study assessed the main features of NSS in the general population, in schizophrenia patients with different onset ages and in their first-degree relatives. Relatives of schizophrenia patients tend to share similar lifestyles, environmental risk factors and genetic factors with the family member who developed schizophrenia. Either environmental risk factors or genetic factors could contribute in varying degrees to familial aggregation of NSS in patients and their first-degree relatives [44]. Past research has also shown that NSS may be influenced by genetic factors and can be the result of a prenatal environmental event. NSS thus represent a marker for schizophrenia risk. NSS are also markers of delays in or slowing brain maturation. Therefore, NSS can be used as endophenotype markers for schizophrenia susceptibility, especially for EOS patient families.

We carried out a comprehensive evaluation of NSS by treating NSS scores as discrete data. Significant poorer performance of NSS could be present in either EOS or AOS patient families than the healthy controls. In past neurodevelopmental models, the genes for schizophrenia susceptibility and chromosomal abnormalities were associated with EOS and resulted in severe premorbid neurodevelopmental abnormalities [45]. The current study obtained the each NSS subscale. In the four NSS subscales, SI and MC had higher recurrence risk ratios than others, and the families of EOS patients tended to have higher recurrence risk ratios than those of AOS patients. Thus, EOS patient families have higher familial aggregation on the SI and MC subscales of NSS compared with AOS patient families.

There are a few limitations of this study. Foremost, patients in this study were not first-episode patients. Several studies have reported confirmed decreases in NSS during neuroleptic treatment in both drug-naïve and first-episode patients [9] as well as follow-up patients [46]. These reports indicate that NSS comprise both state- and trait-features. However, since treatment does not seem to affect NSS scores, it would be ideal to include drug-naïve patients in future studies to eliminate the influence of antipsychotic medications. Second, the inclusion of younger relatives of schizophrenia patients who had not passed the onset age of schizophrenia, especially siblings and children at high risk for schizophrenia in the prodromal state [47] may have led to case status misclassifications. Finally, the onset age information of most schizophrenia patients was available from medical records. However, a potential recall bias could exist because a few participants’ self-reported onset age was used when the chart was unavailable.

The findings of this study were that NSS models had higher discriminant validity for EOS patients than for AOS patients. These results indicate that NSS in EOS patients have more rendered regions than in AOS patients. Importantly, it seems that EOS patients may specifically have severe language processing impairments that are detectable with the SI and MC NSS subscales. Furthermore, EOS patient families have more familial aggregation than AOS patient families in terms of sensory integration and motor coordination. It is likely that there are greater genetic effects on relatives of EOS patients. In summary, the findings support NSS models should be useful to discriminate between early-onset and adult-onset schizophrenia.

## Figures and Tables

**Table 1:** Demographic characteristics of schizophrenia patients with different onset ages, non-psychotic relatives and healthy controls.

	Schizophrenia Patients						Non-psychotic Relatives						Controls	
Variable	EOS (N = 82)		AOS (N = 123)		Total (N = 205)		Relatives of EOS (N = 53)		Relatives of AOS (N = 81)		Total (N = 134)		(N = 243)	
	N	%	N	%	N	%	N	%	N	%	N	%	N	%
Male	52 ^a^	63.40	84	68.29	136	66.34	26	49.12	27	33.33	53	39.55	102	41.98
	Mean	SD	Mean	SD	Mean	SD	Mean	SD	Mean	SD	Mean	SD	Mean	SD
Onset age (yrs.)	16.96 ^a^	1.83	27.12	6.35	23.70	7.56	-	-	-	-	-	-	-	-
Age (yrs.)	36.85	9.91	43.59	9.19	41.89	9.58	55.47	13.18	55.80	15.45	56.52	14.97	42.10	10.95
Weight (kg)	71.61	16.45	67.94	14.81	69.45	15.58	61.36	11.00	63.12	12.11	62.29	11.62	65.32	13.37
Height (cm)	165.49	8.31	165.14	11.76	165.10	10.32	161.16	8.04	160.28	7.88	160.16	8.03	163.41	7.93
BMI	26.01	4.81	24.92	4.87	25.46	4.85	23.60	3.74	25.10	4.31	24.40	3.94	24.37	4.19
Duration of illness (yrs.)	19.88	9.69	16.01	9.47	17.11	9.68	-	-	-	-	-	-	-	-
DIGS-C Positive scale ^‡^	50.65	34.95	48.58	30.78	49.41	44.62	-	-	-	-	-	-	-	-
DIGS-C Negative scale ^‡^	61.59	29.88	58.77	29.12	59.90	42.11	-	-	-	-	-	-	-	-

EOS, early-onset of schizophrenia; AOS, adult-onset of schizophrenia; Total, all schizophrenia patients; SD, standard deviation; BMI, body-mass index; DIGS, Diagnostic Interview for Genetic Studies. ^a^ significant difference between EOS and AOS, *p* < 0.05. ^‡^ Frequency matching was used in EOS and AOS groups by DIGS-C positive and negative score.

**Table 2:** Comparison of NSS scores for patients with early-onset and adult-onset schizophrenia versus healthy controls.

	EOS (N = 82)	AOS (N = 123)	HC (N = 243)	*P*-Value		
NSS Scores				EOS vs. HC	AOS vs. HC	EOS vs. AOS
**NSS total scores**						
Median (range)	8 (0–32)	7 (0–31)	1 (0–8)			
Mean (SD)	9.95 (6.91)	7.58 (5.44)	1.43 (1.69)	<0.001	<0.001	<0.001
**Sensory integration** subscale						
Median (range)	1 (0–7)	0 (0–4)	0 (0–2)			
Mean (SD)	1.43 (1.71)	0.59 (0.85)	0.15 (0.43)	<0.0001	<0.0001	0.0001
**Motor coordination** subscale						
Median (range)	0 (0–10)	0 (0–4)	0 (0–1)			
Mean (SD)	0.93 (1.69)	0.38 (0.54)	0.02 (0.13)	<0.0001	<0.0001	0.0002
**Sequencing of complex motor acts** subscale						
Median (range)	2 (0–7)	2 (0–7)	0 (0–4)			
Mean (SD)	2.17 (1.84)	1.99 (1.69)	0.43 (0.68)	<0.0001	<0.0001	0.15
**Others** subscale						
Median (range)	4 (0–13)	3 (0–11)	0 (0–4)			
Mean (SD)	4.26 (2.97)	3.10 (1.87)	0.43 (0.68)	<0.0001	<0.0001	0.001

NSS, neurological soft signs; EOS, early-onset of schizophrenia; AOS, adult-onset of schizophrenia; HC, healthy controls; SD, standard deviation.

**Table 3:** Performance comparison of NSS-based data mining models for training full data and 10-fold cross-validation results.

	EOS			AOS			Total		
Variable	ANN	DT	LR	ANN	DT	LR	ANN	DT	LR
**NSS subscale**									
Sensory integration	*	*	*	*	*	*	*		*
Motor coordination	*	*	*	*	*	*	*	*	*
Sequencing of complex motor acts	*		*	*		*	*	*	*
Other		*			*	*		*	*
***Training Full Data***									
AUC	0.88	0.87	0.85	0.84	0.85	0.82	0.87	0.86	0.84
Accuracy	0.84	0.83	0.82	0.78	0.80	0.78	0.82	0.82	0.79
Sensitivity	0.80	0.77	0.80	0.73	0.83	0.79	0.74	0.85	0.79
Specificity	0.87	0.87	0.86	0.85	0.81	0.78	0.88	0.83	0.80
***10-fold Cross-Validation***									
AUC	0.87	0.85	0.85	0.83	0.81	0.81	0.86	0.85	0.84
Accuracy	0.84	0.82	0.81	0.78	0.80	0.76	0.82	0.81	0.76
Sensitivity	0.84	0.82	0.82	0.79	0.75	0.77	0.75	0.83	0.78
Specificity	0.86	0.84	0.77	0.78	0.85	0.76	0.88	0.84	0.76

NSS, neurological soft signs; EOS, early-onset of schizophrenia; AOS, adult-onset of schizophrenia; Total, all schizophrenia patients. ANN, artificial neural networks; DT, decision trees; LR, logistic regression; AUC, area under the curve. * Variables entered for each method after feature selection.

**Table 4:** Comparison of NSS scores for non-psychotic relatives of early-onset and adult-onset schizophrenia versus healthy controls.

	Relatives of EOS (N = 53)	Relatives of AOS (N = 81)	HC (N = 243)	*P*-Value		
NSS Scores				REOS vs. HC	RAOS vs. HC	REOS vs. RAOS
**NSS total scores**						
Median (range)	4 (0–18)	4 (0–19)	1 (0–8)			
Mean (SD)	4.51 (4.0)	4.87 (3.8)	1.43 (1.69)	<0.001	<0.001	0.452
**Sensory integration** subscale						
Median (range)	0 (0–3)	0 (0–3)	0 (0–2)			
Mean (SD)	0.34 (0.71)	0.39 (0.75)	0.15 (0.43)	<0.0001	<0.0001	0.912
**Motor coordination** subscale						
Median (range)	0 (0–3)	0 (0–1)	0 (0–1)			
Mean (SD)	0.45 (0.90)	0.41 (0.72)	0.02 (0.13)	<0.0001	<0.0001	0.791
**Sequencing of complex motor acts** subscale						
Median (range)	1 (0–8)	1 (0–4)	0 (0–4)			
Mean (SD)	1.43 (1.62)	1.54 (1.57)	0.43 (0.68)	<0.0001	<0.0001	0.882
**Others** subscale						
Median (range)	2 (0–7)	2 (0–8)	0 (0–4)			
Mean (SD)	2.34 (2.09)	2.60 (1.98)	0.43 (0.68)	<0.0001	<0.0001	0.479

NSS, neurological soft signs; EOS, early-onset of schizophrenia; AOS, adult-onset of schizophrenia; HC, healthy controls; REOS, relatives of EOS; RAOS, relatives of AOS; SD, standard deviation.
